# *Giardia* fatty acyl-CoA synthetases as potential drug targets

**DOI:** 10.3389/fmicb.2015.00753

**Published:** 2015-07-22

**Authors:** Fengguang Guo, Guadalupe Ortega-Pierres, Raúl Argüello-García, Haili Zhang, Guan Zhu

**Affiliations:** ^1^Department of Veterinary Pathobiology, College of Veterinary Medicine and Biomedical Sciences, Texas A&M University, College Station, TexasUSA; ^2^Department of Genetics and Molecular Biology, Center for Research and Advanced Studies of the National Polytechnic Institute, Mexico CityMexico

**Keywords:** *Giardia intestinalis*, fatty acyl-CoA synthetases, drug target, protein modeling, triacsin-C

## Abstract

Giardiasis caused by *Giardia intestinalis* (syn. *G. lamblia*, *G. duodenalis*) is one of the leading causes of diarrheal parasitic diseases worldwide. Although limited drugs to treat giardiasis are available, there are concerns regarding toxicity in some patients and the emerging drug resistance. By data-mining genome sequences, we observed that *G. intestinalis* is incapable of synthesizing fatty acids (FA) *de novo*. However, this parasite has five long-chain fatty acyl-CoA synthetases (GiACS1 to GiACS5) to activate FA scavenged from the host. ACS is an essential enzyme because FA need to be activated to form acyl-CoA thioesters before they can enter subsequent metabolism. In the present study, we performed experiments to explore whether some GiACS enzymes could serve as drug targets in *Giardia*. Based on the high-throughput datasets and protein modeling analyses, we initially studied the GiACS1 and GiACS2, because genes encoding these two enzymes were found to be more consistently expressed in varied parasite life cycle stages and when interacting with host cells based on previously reported transcriptome data. These two proteins were cloned and expressed as recombinant proteins. Biochemical analysis revealed that both had apparent substrate preference toward palmitic acid (C16:0) and myristic acid (C14:0), and allosteric or Michaelis–Menten kinetics on palmitic acid or ATP. The ACS inhibitor triacsin C inhibited the activity of both enzymes (IC_50_ = 1.56 μM, *K*_i_ = 0.18 μM for GiACS1, and IC_50_ = 2.28 μM, *K*_i_ = 0.23 μM for GiACS2, respectively) and the growth of *G. intestinalis in vitro* (IC_50_ = 0.8 μM). As expected from giardial evolutionary characteristics, both GiACSs displayed differences in overall folding structure as compared with their human counterparts. These observations support the notion that some of the GiACS enzymes may be explored as drug targets in this parasite.

## Introduction

*Giardia intestinalis* (syn. *G. lamblia*, *G. duodenalis*) is one of the major causative agents of diarrheal diseases in humans around the world. In the U.S. alone, there are estimated 1.2–2 million (but up to 8 million) cases per year, resulting in annual costs of >$30 million USD (Hlavsa et al., 2005; Yoder et al., 2007, 2010, 2012). The recently reported infection rates range from 0.4–7.6% in developed countries, and 0.9–40% in developing countries (Plutzer et al., 2010; Feng and Xiao, 2011). Because *Giardia* cysts exhibit moderate resistance to the chlorine used in treating water for drinking and recreational purposes, it is also one of the most common water-borne pathogens and is listed as a Category B priority pathogen in the NIH/CDC Biodefense program^1^. In addition to water-borne and food-borne transmissions, *G. intestinalis* is also a zoonotic pathogen capable of transmitting infections between animals and humans. Drugs to treat giardiasis are available, but the choices are limited (e.g., metronidazole, tinidazole, albendazole, and nitazoxanide) (Busatti et al., 2009; Tian et al., 2010; Tejman-Yarden and Eckmann, 2011; Granados et al., 2012). These drugs are also not 100% effective and may be unsuitable for some patients due to toxicity. Drug resistance is also an emerging problem (Ali and Nozaki, 2007; Lalle, 2010; Tian et al., 2010). Therefore, new or alternative drugs are needed, particularly if massive infection occurs under natural or man-made conditions.

**FIGURE 1:**
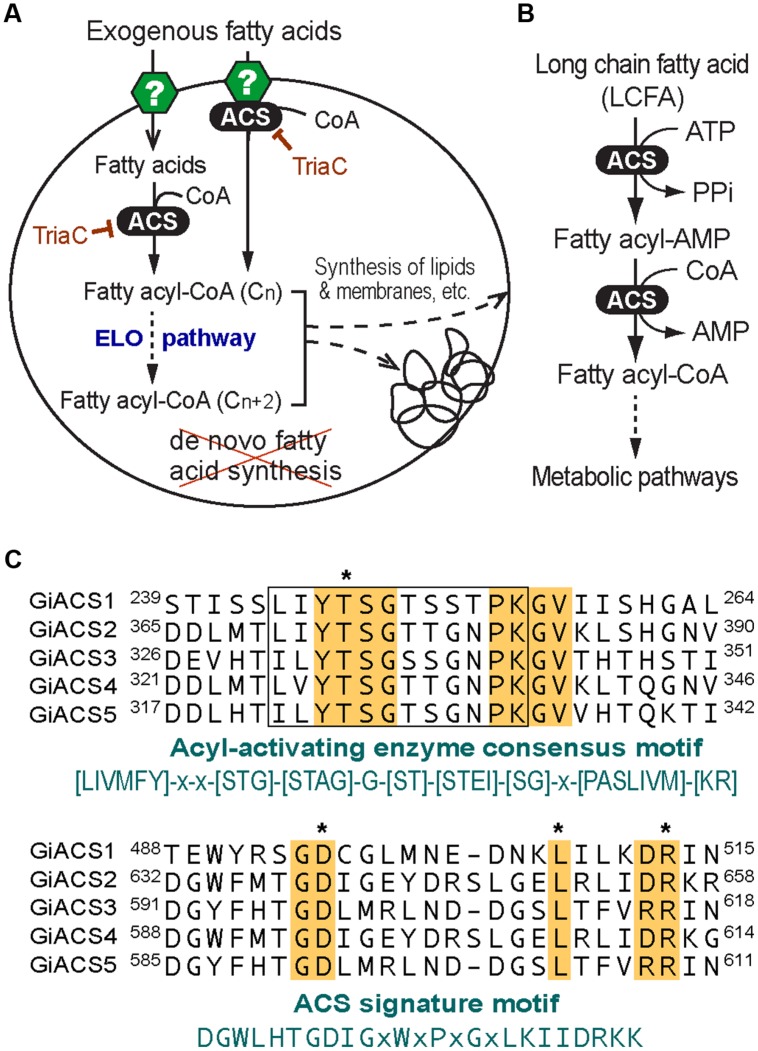
**Fatty acid (FA) metabolism and acyl-CoA synthetase (ACS) in *Giardia intestinalis*. (A)** Highly streamlined FA metabolism in *G. intestinalis* based on the genome sequences. This parasite relies on exogenous FA due to the incapability of synthesizing FA *de novo*. FA are transported into parasite cell by undefined transporters or other mechanisms. FA are activated by ACS immediately or from the FA pool to form FA-CoA thioesters that may be elongated via the ER-associated elongation system (ELO) table before entering subsequent synthetic pathways. The ACS activity may be inhibited by triacsin C. **(B)** ACS catalyzes a two-step reaction to form FA-CoA thioesters from FA and CoA; **(C)** Multiple alignments of the five GiACS proteins at the AMP-binding domain conserved in acyl-activating enzymes and ACS signature motif. Residues conserved in all GiACSs are shaded, while those important to the catalytic function and interacting substrates are boxed and marked with asterisks, respectively. Also, see Supplementary Figure S1 for a multiple alignment of the five full-length GiACS protein sequences with annotation of predicted active sites.

*Giardia* species are anaerobic protozoa evolutionarily branched early at the base of eukaryotes (Morrison et al., 2007). The genome of *G. intestinalis* has been sequenced and reported in 2007, which revealed that this parasite has none or limited ability to synthesize most nutrients *de novo*, such as amino acids, nucleosides, and fatty acids (FA) (Morrison et al., 2007). In FA metabolism, *Giardia* lacks both types I and II synthetic pathways, and thus relies on scavenging FAs from hosts. This notion is also supported by earlier biochemical analysis on this parasite (Jarroll et al., 1981; Beach et al., 1990; Das et al., 2002; Lee et al., 2007). This anaerobic protozoan retains limited fatty acyl extension ability by possessing one or more elongating (*ELO*) genes. Congruent with its anaerobic life style, *Giardia* also lacks enzymes for FA degradation and β-oxidation. FA scavenged from hosts are first activated by acyl-CoA synthetase (ACS, aka. FA-CoA ligase, ACL) to form fatty acyl-CoA (FA-CoA) thioesters before they can enter to subsequent metabolic pathways, such as FA elongation and synthesis of lipids and biomembranes (**Figures 1A,B**). Therefore, targeting ACS may block the entire FA metabolism, thus killing the parasite.

In this study, we cloned and expressed two of the five ACSs from *G. intestinalis* (named as GiACS1 and GiACS2) as maltose-binding protein (MBP)-fusion proteins, and characterized their substrate preference and enzyme kinetic features. We also showed that the ACS inhibitor triacsin C could not only inhibit the activity of GiACS1 and GiACS2, but also display efficacy against the growth of *G. intestinalis in vitro* at micromolar levels.

## Materials and Methods

### Data-mining the *G. intestinalis ACS* Genes and their Expression Profiles

To ensure the full recovery of *ACS* genes from the *G. intestinalis* genomes, we searched the *Giardia*DB^2^ and the *Giardia* reference genomes at the National Center for Biotechnology Information^3^ (NCBI) with relevant keywords and by BLAST searches using known long-chain fatty acyl (LCFA)-CoA protein sequences as queries. The identities of GiACS proteins were further confirmed by BLAST searches for their orthologs and signature domains at the NCBI Conserved Domain Database^4^ (CDD). This strategy identified five *G. intestinalis* ACS (*GiACS*) orthologs and three related genes that are summarized in **Table 1**.

**Table 1:** Fatty acyl-CoA (FA-CoA) synthetase (ACS) orthologs and related genes identified from the *Giardia intestinalis* genome and their top hits at the NCBI conserved domain (CDD) database.

Gene Name	GenBank Accession No.	*Giardia*DB Gene ID	*Giardia*DB description	Protein size	CDD top hit	CDD accession	*E*-value to CDD top hit
*GiACS1*	XP_001705891	GL50803_9062	Long-chain fatty acid (FA) CoA ligase 5	853 aa	LC-FACS_euk	cd05927	8.73E-120
*GiACS2*	XP_001706424	GL50803_15063	Long-chain FA CoA ligase 5	693 aa	LC-FACS_euk	cd05927	1.92E-157
*GiACS3*	XP_001705009	GL50803_21118	Long-chain FA CoA ligase 5	765 aa	LC-FACS_euk	cd05927	2.72E-117
*GiACS4*	XP_001708520	GL50803_30476	Long-chain FA CoA ligase 4	804 aa	LC-FACS_euk	cd05927	8.47E-163
*GiACS5*	XP_001709411	GL50803_113892	Long-chain FA CoA ligase, putative	758 aa	LC-FACS_euk	cd05927	5.37E-117
Unnamed	XP_001707853	GL50803_17170	Long-chain FA CoA ligase 5	1,523 aa	VL_LC_FACS_like	cd05907	6.85E-19
Unnamed	XP_001710279	GL50803_86511	Acyl-CoA synthetase (ACS)	970 aa	ATP-grasp_5	pfam13549	4.05E-38
Unnamed	XP_001709605	GL50803_16667	ACS	905 aa	ATP-grasp_5	pfam13549	1.16E-38

By taking advantage of already published and publically available transcriptome datasets at the *Giardia*DB, we also data-mined the expression levels and fold changes of the five *GiACS* genes to evaluate their importance and potential differential roles in various parasite stages. These included their transcript levels in trophozoites and cysts, as well as during the encystation, excystation, and interactions with host cells that were determined by serial analysis of gene expression (SAGE), microarray analysis, and RNA-seq using the Illumina HiSeq2000 platform (Palm et al., 2005; Morf et al., 2010; Ringqvist et al., 2011; Franzen et al., 2013). Expression data of individual GiACS genes were extracted from corresponding datasets in *Giardia*DB and values were plotted for comparison.

### Protein Modeling

Structure homology modeling was performed on GiACS1 and GiACS2 (853 and 693 aa, respectively) using the I-Tasser (**I**terative **T**hreading **ASSE**mbly **R**efinement) webserver at http://zhanglab.ccmb.med.umich.edu/I-TASSER/ (Zhang, 2008; Roy et al., 2010). This platform is the most widely used system to build structural protein models from query sequences using the solved crystal structures contained at the RCSB Protein Data Bank (PDB) as templates. The quality of the model is given by a C-score (range –5 to 2), which is an index that considers TM and RMSD scores and allows for the ranking of the degrees of similarity between query and template protein structures. The C-score is used in combination with the TM score (range 0–1) to obtain the best model (Yang et al., 2015). The root-mean-square-deviation (RMSD) score indicates a measure of the differences (in Å) between values predicted by retrieved models and the values actually observed in PDB templates. The quality of protein models obtained was further visualized and tested by Ramachandran plots in the Discovery Studio v4.1 client (Accelrys^TM^) software. To compare the overall folding of two given protein models, the PDB files generated by I-Tasser platform were submitted to the TM-Align platform^5^ that retrieves the RMSD and TM scores for the structural alignment of the proteins studied. According to PDB statistics, TM-scores below 0.2 corresponds to randomly chosen unrelated proteins, whereas a score higher than 0.5 match generally the same fold.

### Expression of Recombinant GiACS Proteins

From identified *GiACS* genes, we chose to first clone and express two genes for potential functional analysis (i.e., *GiACS1* and *GiACS2*, corresponding to the *G. intestinalis* WB strain Gene ID numbers GL50803_9062 and GL50803_15063, or GenBank accession numbers XP_001705891 and XP_001706424, respectively) (**Table 1**). Genomic DNA was isolated from the WB strain of *G. intestinalis* (ATCC # 30957) using Qiagen DNeasy Blood & Tissue Kit using protocol recommended for cultured cells. For biochemical analysis, the entire intron less open reading frames (ORFs) of *GiACS1* and *GiACS2* genes were amplified from the genome DNA by PCR using high-fidelity *Pfu* Turbo HotStart DNA polymerase (Agilent Technologies, Los Angeles, CA, USA). Linker sequences containing *Bam*HI and *Hind*III restriction sites were added to the sense and antisense primers, respectively (**Table 2**). The PCR products were digested by *Bam*HI and *Hind*III, and ligated into the linearized pMAL-c2E vector. The ligated plasmids were transferred into the Rosetta 2 strain of *Escherichia coli* competent cells (Novagen) and cultured in LB agar plates containing 100 μg/mL ampicillin, from which plasmids were isolated from individual colonies by E.Z.N.A. plasmid DNA miniprep kit (Omega Bio-Tek, Atlanta, GA, USA) and sequenced by Sanger sequencing technique at the Texas A&M University Gene Technologies Laboratory^6^ to confirm their identity and sequence accuracy.

**Table 2:** Primers used in the cloning of *GiACS1* and *GiACS2* genes.

Gene Name	Orientation	Linker	Sequence (5′–3′)^1^
*GiACS1*	Forward	*Bam*HI	ctggatccATGATCTTTCCATTTCTAAAAC
*GiACS1*	Reverse	*Hind*III	gcaagcttCTCTCCTTATCAACCATGGCTTC
*GiACS2*	Forward	*Bam*HI	ctggatccATGTCGGATTTCATCTGCC
*GiACS2*	Reverse	*Sal*I	gcgtcgacCTTACTAGATGGTCTAGA

*^1^Lower cases indicate added linker sequences.*

The expression of MBP-fusion proteins was carried out as described (Guo and Zhu, 2012; Guo et al., 2014; Yu et al., 2014). Briefly, engineered plasmids were transferred into the Rosetta 2 strain of *E. coli* to grow colonies in LB agar plates containing 100 μg/mL ampicillin and 34 μg/mL chloramphenicol, from which bacterial colonies (less than 1 week old) were individually transferred into 25 mL LB broth containing 0.2% glucose and allowed to grow at 37°C overnight. The next day, bacterial broths were diluted by 1:100 with fresh medium and incubated at 37°C for 2 h or until OD_495_ reached to ∼0.5, followed by the induction of expression by isopropyl β-D-1-thiogalactopyranoside (IPTG) (0.3 mM) at 16°C overnight. Bacteria were collected by centrifugation (6000 × *g*, 10 min) and lysed by sonication in Tris-HCl buffer (pH 7.5), from which the recombinant proteins were purified by amylose resin-based affinity chromatography according to the manufacturer’s instructions (New England Biolabs). The quality and quantity of purified proteins were analyzed by SDS-PAGE and Bradford assay using BSA as the protein standard. Proteins were used immediately after the purification or stored at –20°C.

### Biochemical Assays

The ACS activity was determined by a five, 5′-dithio-bis-(2-nitrobenzoate) (DTNB) colorimetric assay for both GiACS1 and GiACS2. In the assay, free CoA in reduced form (CoA-SH) reacted with DTNB to form 5-thionitrobenzoic acid that was measured at OD_412_ (Bernson, 1976; Zhuravleva et al., 2012; Guo et al., 2014). Reactions were carried out in 200 μL Tris-HCl buffer (0.1 mM, pH 8.0) containing 10 mM KCl, 50 μM CoA, 500 μM ATP, 10 mM MgCl_2_, and 100 μM FA. The concentrations of substrates and cofactors might be varied for determining their dose-response curves and FA with carbon chains ranging from C2:0 to C30:0 were used for determining substrate preference. Reactions were started by the addition of 1 μg of recombinant MBP-GiACS proteins, followed by incubation at 32°C for 10 min, and then stopped by heating samples at 80°C for 5 min. Following sample cooling to room temperature, 4 μL of DTNB (5 mM) was added into each reaction that was allowed for color development for 5 min, followed by the measurement of OD_412_ values with a Multiskan Spectrum spectrophotometer (Thermo Scientific). Serial concentrations of CoA in the same reaction buffer were assayed and used as standard curves for calculating the amounts of CoA reduced in experimental samples. Enzyme kinetic parameters were determined using varied concentrations of palmitic acid (10–600 μM) and ATP (10– 3000 μM), respectively.

To confirm the formation of palmitoyl-CoA catalyzed by GiACS1 and GiACS2, we performed a radioactive assay in which reactions were carried out with the use of ^3^H-palmitic acid (25 μM) and other reagents as described above. Negative controls consisted of an MBP-tag to replace MBP-GiACS proteins for background subtraction. After the reaction, samples were subjected to a heptane extraction procedure to remove free palmitic acid, and the radioactivity of ^3^H-palmitoyl-CoA in the aqueous phase was counted in a Beckman Coulter LS 6000SE counter as described (Fritzler and Zhu, 2007; Fritzler et al., 2007). We also evaluated the effects of an ACS inhibitor, triacsin C (2,4,7-undecatrienal nitrosohydrazone; CAS 76896-80-5) on the GiACS activity, in which 1–32 μM of triacsin C was used to determine the IC_50_ value. In each experiment, there was a set of reactions under the same conditions, but without enzyme for use as controls for background subtractions.

### Efficacy of Triacsin C on the Growth of *G. intestinalis In Vitro*

The effect of triacsin C on the growth of *G. intestinalis* (WB strain) *in vitro* was assessed by subculture in liquid medium as described (Arguello-Garcia et al., 2004). In this assay, 1 × 10^6^ *Giardia* trophozoites were cultured in 4.5-mL screw-capped vials containing fresh TYI-S-33 medium (less than 1 week old) containing varied concentrations of Triacsin C (0.13–16 μM) for 24 h at 37°C. Then 1 × 10^5^ drug-exposed trophozoites were transferred to new 4.5-mL vials containing drug-free medium and incubated for additional 48 h at 37°C. Parasites were then counted and the parasite growth was expressed as the percent of surviving trophozoites in comparison to those in the negative controls that did not received an inhibitor.

## Results

### The Genome of *G. intestinalis* Encodes Five Long Chain FA-CoA Synthetases that are Differentially Expressed the Parasite

By data-mining the genome sequences of *G. intestinalis* (WB), we identified eight candidate genes encoding proteins that either exhibited high degree of identities with other known ACS proteins or annotated as long-chain FA CoA ligases or ACSs by the *Giardia*DB (**Table 1**). Among them, five genes (designated as *GiACS1* to *GiACS5*) appear to encode for ACS enzymes based on their identities to other ACS proteins and by the presence of AMP-binding domain, ACS signature motifs, and other active sites in their protein sequences (**Figure 1C**; Supplementary Figure S1). The top hit at the NCBI CDD for all five GiACS proteins is LC_FACS_euk (CDD No. cd05927) with expectation values (*E*-values) ranging from 1.92E-157 to 5.37E-117 (**Table 1**). Among the other three genes, GL50803_17170 (GenBank: XP_001707853) was annotated as “LCFA CoA ligase 5,” and several short regions within the sequence could be mapped to the “VL_LC_FACS_like” domain at CDD (cd05907) with a much less significant E-value at 6.85E-19 (**Table 1**). The sequence also lacked most of the active sites in ACS, but only contained limited conserved amino acids at the two signature motifs, suggesting that GL50803_17170 was derived from an ancient very-long LC-FACS, but probably lost its ACS function. The remaining two genes were annotated as ACSs (GL50803_86511, GenBank: XP_001710279; and GL50803_16667, GenBank: XP_001709605) (**Table 1**). They both contained an ATP_grasp-5 domain (CDD: pfam549) with less significant *E*-values at 4.05E-38 and 1.16E-38, respectively. Interestingly, proteins encoded by these two genes were ortholog of CoA-binding proteins from prokaryotes, suggesting they were derived from prokaryotic genes by lateral gene transfer and might more likely function as CoA-binding proteins than as ACS. Based on these observations, we concluded that the *G. intestinalis* genome encoded five ACSs and three ACS-related proteins.

**FIGURE 2:**
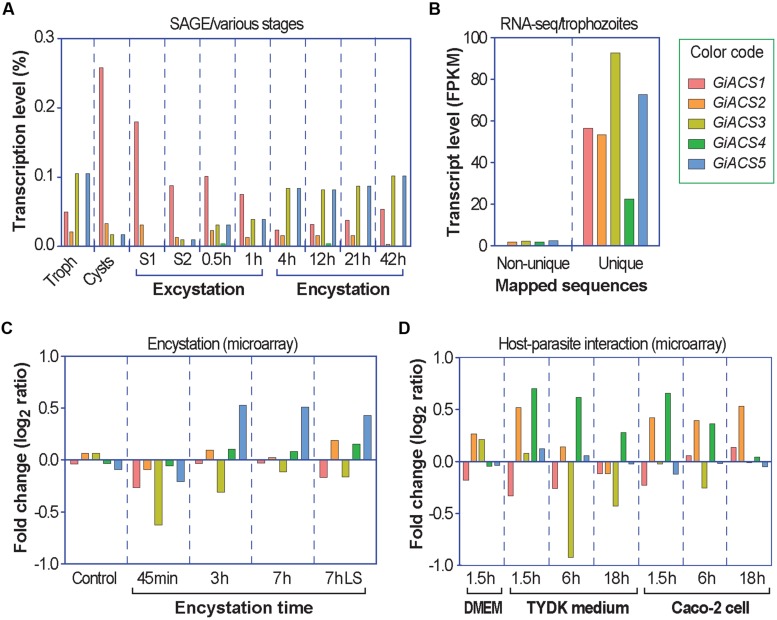
**Differential expressions of the five *GiACS* genes as determined by data-mining previously published transcriptome data available at *Giardia*DB**. All data were derived from *G. intestinalis* assemblage A WB strain. **(A)** Expression profiles based on serial analysis of gene expression (SAGE) in trophozoites (Troph), cysts, and during excystation and encystation (Palm et al., 2005). S1 (stage 1) = under acidic condition to mimic the stomach. S2 (stage 2) = trypsin and slight alkaline conditions to mimic the small intestines. **(B)** Transcriptional levels in trophozoites based on strand-specific RNA-seq using the Illumina HiSeq2000 platform (Franzen et al., 2013). FPKM = fragments per kilobase of exon model per million mapped reads; **(C)** Transcriptional changes in response to encystation stimuli based on a dual-color hybridization of a full-genome microarray analysis, in which encystation was induced *in vitro* for 45 min, 3 h, and 7 h by standard 2-step protocol or for 7 h by lipid starvation (7hLS) (Morf et al., 2010). **(D)** Transcriptional changes in trophozoites during interacting with host cells as determined by a full-genome microarray analysis, in which trophozoites were used to infect Caco-2 cells in DMEM medium or cultured in cell-free TYDK medium for varied times (Ringqvist et al., 2011).

Further transcriptome analysis by data-mining previously published transcriptome data generated using various platforms (i.e., SAGE, RNA-seq, and microarray) and available at *Giardia*DB (Palm et al., 2005; Morf et al., 2010; Ringqvist et al., 2011; Franzen et al., 2013), we noticed that the five *GiACS* genes were differentially expressed in different parasite stages (**Figure 2**). In trophozoites, an earlier SAGE analysis detected the transcripts of four *GiACS* genes (with the exception of only *GiACS4*) (**Figure 2A**), whereas a more recent RNA-seq analysis was able to detect the transcripts of all five *GiACS* genes (**Figure 2B**). However, the general expression profiles in these two datasets were similar (i.e., the highest for *GiACS3* and *GiACS5*, moderate for *GiACS1* and *GiACS2*, and the lowest for *GiACS4*). In other stages including cysts and parasites during encystation and excystation, *GiACS1* was the most highly expressed among all *GiACS* genes in all stages in the SAGE dataset (**Figure 2A**). *GiACS1* also maintained the highest levels of expression during excystation process, but mid-level expressions during the encystation process (**Figure 2A**). *GiACS2* was consistently expressed at moderate levels in all parasite stages, while *GiACS3* had relatively high transcript levels in trophozoites and during encystation, but generally lower levels in cysts and excysation. *GiACS4* was consistently the lowest or undetectable in all stages, whereas *GiACS5* was highly expressed in trophozoites and during encystation, but slightly expressed during excystation (**Figure 2A**). In agreement with the SAGE analysis, a microarray analysis also detected apparent down-regulation of *GiACS3* and up-regulation of *GiACS5* during the encystation process (**Figure 2C**). Although *GiACS4* gene displays the lowest or even undetectable expression levels in all parasite life cycle stages, this gene significantly elevated its expression during the interaction with host cells or when culture medium is changed from DMEM to TYDK medium (**Figure 2D**). Although further functional studies are needed to delineate the biological roles played by the five *Giardia ACS* genes, their differential expressions in various biological processes and conditions clearly imply that they might play differential roles in the parasite life cycle.

### Insights from GiACS1 and GiACS2 Protein Models

Considering the aforementioned data and the fact that chemotherapy in giardiasis is mostly directed against the trophozoite stage, the GiACS1, and GiACS2 proteins were considered as potential drug targets due to their stable and comparable transcription levels in trophozoites (**Figure 2B**), even though expression of both proteins differ upon trophozoite-epithelial cell interactions. GiACS2 was up regulated while GiACS1 was down regulated at ≤ 6 h of interacting with host cells (**Figure 2D**). In addition, GiACS1 is a much larger protein (853 aa) than the other GiACSs and their human counterparts (generally <700 aa); hence, the GiACS1 represents a divergent ACS.

**FIGURE 3:**
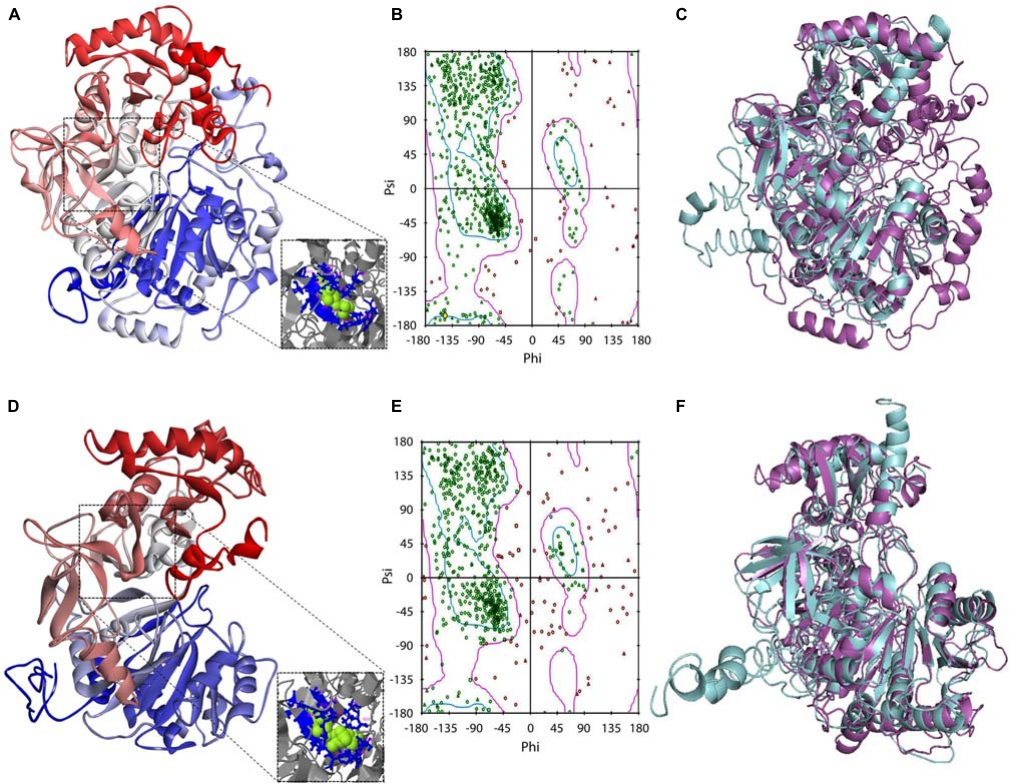
**Protein modeling features of GiACS1 and GiACS2**. The protein folding models for GiACS1 **(A)** and GiACS2 **(D)** were retrieved using yeast ACS (PDB ID: 1RY2) as a template. Protein models are colored from N-terminus (blue) to C-terminus (red) and oriented in relation to the AMP/ATP heterogen (insets). The Ramachandran plots calculated for GiACS1 **(B)** and GiACS2 **(E)** display glycine (triangles), proline (squares), and non-glycine non-proline (circles) residues. The structural alignments of GiACS1 (magenta) with HsACS5CRAc (cyan) **(C)** and 541 GiACS2 (magenta) with HsACS5b (cyan) **(F)** display folding differences between giardial and human counterparts.

In an initial mining of ACS crystals in PDB, protozoan templates were absent. When the GiACS1 and GACS2 sequences were submitted to preliminary modeling, the retrieved models shared the highest scores with members of the CoA synthetase family as firefly luciferase, ACS of *Saccharomyces cerevisiae* and acetoacetyl-CoA synthetase of *Streptomyces lividans* (PDB IDs: 2D1S, 1RY2, and 4WD1, respectively). From these, the yeast homolog (1RY2) was chosen as template for refinement of protein models due to its ACS nature that closely matches with the proposed GiACS functions. In this way, a template-directed modeling was performed and the two GiACS models (**Figures 3A,D**) had satisfactory scores: C-score: –2.57, TM-score = 0.636, and RMSD = 0.95 for GiACS1; *C*-score: –1.04, TM-score = 0.773, and RMSD = 1.65 for GiACS2. These data reflect that GiACS models share 63.6% (GiACS1) and 77.3% (GiACS2) homology (average deviation <2Å) with respect to the yeast ACS template. Also, the identity in sequence may be indicated with the available PDB structures resolved since I-Tasser is good for modeling protein targets in the “twilight zone” (20–30% identity), which have no or weakly homologous templates (Roy et al., 2010).

In a further assessment of the quality for GiACS1/2 protein models, the Ramachandran plots were constructed. This tool not only predicts secondary structures from dihedral angles of individual amino acids (φ and ψ), but also provides distributions of residues in “favored regions” (contoured in blue line), “additionally allowed regions” (contoured in pink line), and the external “generously allowed” and “not allowed” regions as stated by the ProCheck platform (**Figures 3B,E**). The special cases of glycine and proline are ruled out as recommended and only residues contained in the two former regions were considered of satisfactory conformation. These distributions for GiACS1 and GiACS2 are listed in **Table 3**. In this, more than 75% of residues fall within favored regions, more than 15% fall within allowed regions and <10% are in external regions. In summary, up to 95.15% of residues in GiACS1 and 89.67% of residues in GiACS2 have a satisfactory conformation within the protein structure predicted for these molecules; hence, the protein models obtained have an acceptable quality.

**Table 3:** Ramachandran plots statistics of GiACS1 and GiACS2.

Residues in Ramachandran plot	GiACS1 (853 aa)	GiACS2 (693 aa)
In most favored regions	581 (78.30%)	443 (72.63%)
In additionally allowed regions	125 (16.85%)	104 (17.04%)
In generously allowed or disallowed regions	36 (4.85%)	63 (10.33%)
Non-Gly and non-Pro residues	742 (100%)	610 (100%)
# Gly (triangles)	66	43
# Pro (squares)	45	40

Further analyses to evaluate GiACS1 and GiACS2 as likely druggable targets were performed to determine the structural alignment of GiACSs with their most resembling human counterparts. In these, two isoforms of the human long chain fatty acyl CoA synthetases had the closest similarity to these GiACSs: the 5CRAc isoform (HsACS5CRAc, Acc. No. gb| EAW49534.1, 663 aa) displaying the highest expectation value (8e-76) and 37% of sequence identity with GiACS1 over a 64.1% coverage and the 5b isoform (HsACS5b, Acc. No. NP_976313.1, 683 aa) that displayed the highest expectation value (5e-60) and 26% of sequence identity with GiACS2 over a 79.4% coverage. Despite the high *E*-values, the structural comparison of giardial and human counterparts revealed striking differences: GiACS1 and HsACS5CRAc had 31.0% of structure identity over a span of 561 aa (RMSD: 3.20; TM-score: 0.769 normalized with HsACS5CRAc) while GiACS2 and HsACS5b shared 23.9% of structure identity over a span of 594 aa (RMSD: 3.90; TM-score: 0.775 normalized with HsACS5b). In general, there were obvious folding differences between the *Giardia* and human ACS counterparts (**Figures 3C,F**), particularly in the case of GiACS1 as a consequence of the multiple insertions contained in this unusually large (853 aa) molecule. Based on these observations, it was tempting to compare purified GiACS in terms of catalytic abilities and susceptibility to specific inhibitors.

### Functional Confirmation of GiACS1 and GiACS2 as a Long Chain FA-CoA Synthetase

**FIGURE 4:**
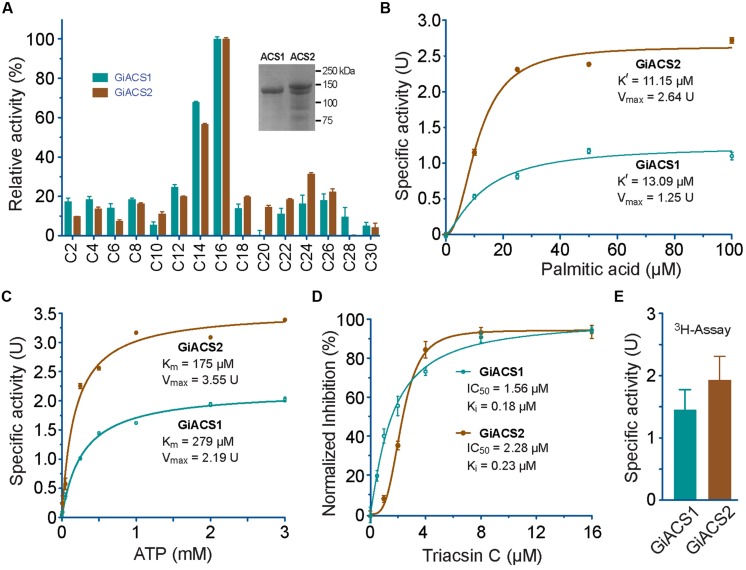
**Enzyme kinetic features of recombinant GiACS1 and GiACS2 as determined by a 5, 5′-dithio-bis-(2-nitrobenzoate) (DTNB) colorimetric assay. (A)** Substrate preference of GiACS1 and GiACS2 toward FA with varied carbon chain lengths. Relative activities were shown in comparison with that on the C16:0 palmitic acid. Inset showed SDS-PAGE analysis of purified recombinant GiACS1 and GiACS2 as maltose-binding protein (MBP)-fusion proteins; **(B)** Allosteric kinetics of GiACS proteins on palmitic acid; **(C)** Michaelis–Menten kinetics of GiACS on ATP; **(D)** Inhibition of triacsin C on the GiACS enzyme activity; and **(E)** Enzyme activity by detecting the formation of ^3^H-palmitoyl-CoA using a heptane extraction-based radioactive assay. Bars represent SEM derived from three or more reactions. At least two independent assays were performed for each experiment, and the data from one representative assay were shown here. *U* = μmol/mg/min.

After we determined that *G. intestinalis* possessed five ACSs, we decided to first investigate the biochemical features for two of them. We selected *GiACS1* and *GiACS2* in our initial study because both genes were relatively highly and consistently expressed in different life cycle stages, and GiACS2 appeared to be important in host–parasite interaction. Their whole ORFs were successfully cloned into the pMAL-c2E expression vector, and their products were expressed as MBP-fusion proteins (**Figure 4A**, inset). GiACS1 protein was purified into high purity, while the majority of GiACS2 was expressed at expected size, but some lower bands were present, suggesting some incomplete translation of GiACS2 probably due to the differences in codon usage between *G. intestinalis* and *E. coli*.

Using DTNB assay, we were able to individually evaluate the activity of GiACS1 and GiACS2 toward various saturated FA with chain lengths ranging from C2:0 to C30:0. Both GiACS1 and GiACS2 displayed the highest activity over palmitic acid (C16:0) and myristic acid (C14:0) (**Figure 4A**). Their activity on other FA was much lower, mostly ranging from ∼5% to ∼25%. These observations confirmed that both GiACS1 and GiACS2 are long-chain FACS with a relatively restricted substrate preference toward C14:0 and C16:0 FA. This feature makes these two GiACS proteins differ from the two *Cryptosporidium* ACSs that could use C12:0–C18:0 FA with comparable levels of activity (Guo et al., 2014). In kinetic studies, these two enzymes exhibited allosteric and Michaelis–Menten kinetics toward palmitic acid and ATP, respectively (**Figures 4B,C**). Their kinetics parameters are listed in **Table 4**. We also validated the function of GiACS1 and GiACS2 by directly detecting the formation of palmitoyl-CoA using a radioactive assay, in which both enzymes exhibited specific activities comparable to those obtained using DTNB assay (**Figure 4E**).

**Table 4:** Kinetic parameters for GiACS1 and GiACS2^1^.

Enzyme	Substrate	*K*′ or *K*_m_ (μM)	*V*_max_ (μmol/mg/min)	*h*^2^	*K*_i_ of triacsin C (μM)
GiACS1	Palmitic acid	13.09 ± 2.42	1.25 ± 0.12	1.36	0.18
	ATP	279 ± 18.46	2.19 ± 0.04		
GiACS2	Palmitic acid	11.15 ± 0.54	2.64 ± 0.06	2.19	0.23
	ATP	175 ± 18.68	3.55 ± 0.08		

*^1^Values for *K*′, *K*_m_, and *V*_max_ are expressed as mean ± SD; ^2^*h* = Hill coefficient.*

### Triacsin C Inhibits GiACS1 and GiACS2 Enzyme Activity as Well as the *In Vitro* Growth of *G. intestinalis*

We further tested whether GiACS1 and GiACS2 were amendable to the inhibition by an ACS inhibitor triacsin C, and observed that this compound could inhibit their enzyme activity with **IC_50_** values at 1.56 and 2.28 μM on GiACS1 and GiACS2, respectively (**Figure 4D**). Their corresponding ***K*_i_** values were at 0.18 and 0.23 μM, respectively, based on a competitive inhibition model (Cheng and Prusoff, 1973) (**Figure 4D**). The efficacy data were comparable to those observed for the two ACSs from the apicomplexan parasite *Cryptosporidium parvum* (i.e., **IC_50_** values at 3.70 and 2.32 μM for CpACS1 and CpACS2, respectively) (Guo et al., 2014).

**FIGURE 5:**
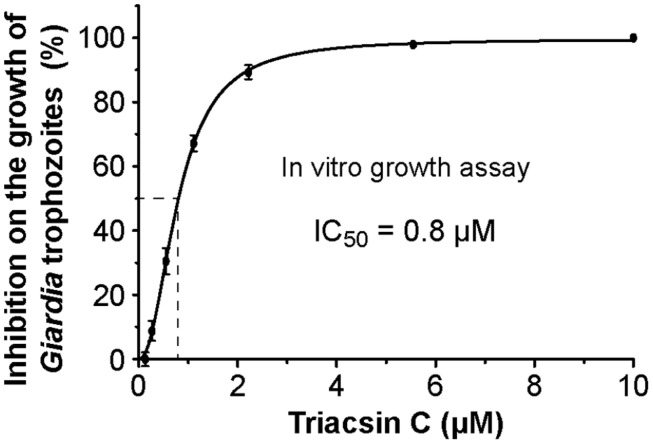
**Efficacy of triacsin C on the growth of *G. intestinalis* (WB strain) *in vitro*, in which axenically cultured trophozoites were treated with various doses of inhibitor for 24 h, transferred to drug-free medium, and allowed to grow for additional 48 h before being counted for calculating the parasite growth**.

Since the major goal of this study is to explore the potential of GiACSs to serve as drug targets, we further investigated the *in vitro* efficacy of triacsin C against the growth of *G. intestinalis* in axenic culture. We observed that triacsin C indeed effectively inhibited the parasite growth in a dose-dependent manner with an estimated IC_50_ value at 0.8 μM (**Figure 5**). At other tested doses, 2.2 μM and ≥10 μM triacsin C achieved 90 and 100% inhibitions, respectively.

## Discussion

FA are essential to all organisms as one of the major components of biomembranes. In most organisms, FA also serves as an energy source. Because FA needs to be activated to form FA-CoA before they can enter subsequent metabolic pathways, enzymes catalyzing the formation of FA-CoA are essential and considered as potential drug targets. We have recently reported that ACS enzymes could serve as effective drug targets in *Cryptosporidium* (Guo et al., 2014), which prompted us to explore whether ACSs could also be targeted for developing novel anti-*Giardia* drugs. Indeed, in the present study, we have confirmed that the ACS inhibitor could inhibit not only the reactions catalyzed by GiACS2, but also the *in vitro* growth of *G. intestinalis* at sub-micromolar levels (**Figure 5**). The possibility to consider GiACS1 and GiACS2 as likely drug targets was further supported by bioinformatics data in which protein modeling analyses showed structural differences between giardial and human counterparts (**Figure 3F**). This fact could be the result of evolutionary processes in which the giardial ACSs are likely ancestors of other ACSs counterparts. In this context, it is conceivable that GiACS1 (853 aa) could have had a reductive process during evolution. In spite of the predicted satisfactory quality of the protein models in this study, further crystallographic studies in purified GiACS1/2 will offer additional insights not only for site-targeted drug design, but also to assess if a differential adaptation in the catalytic pocket of GiACS2, as compared to GiACS1, exists. Moreover, the possibility to recover enzyme activities from recombinant GiACS1/2 will allow refining crystallographic analyses at distinct enzyme conformations and under interaction with Triacsin C or other specific inhibitors. This renders an advantage over the failure to detect enzyme activities in other parasitic recombinant ACSs (Matesanz et al., 1999; Guo et al., 2014).

Triacsin C is an analog of polyunsaturated FA that was first isolated from the fungal *Streptomyces aureofaciens* (Omura et al., 1986). It is known as a long-chain ACS-specific inhibitor with little effect on short-chain or mitochondrial-type ACSs in mammals (Omura et al., 1986; Tomoda et al., 1987; Hartman et al., 1989; Van Horn et al., 2005). Mammals possess six genes encoding long-chain ACS enzymes that are designated as ACSL1-6 including some variants produced by alternative intron-splicing with varied spectra of substrate preferences (Soupene and Kuypers, 2008; Watkins and Ellis, 2012). Among them, triacsin C is an effective inhibitor for ACSL1, ACSL3, and ACSL4 (IC_50_ values between 4 and 7.5 μM), but not for ACSL5, ACS6_v1, and ACS6_v2 (Van Horn et al., 2005). The abundance of ACSLs and their varied sensitivities to triacsin C might explain its ineffectiveness on the recycling of FA into phospholipids in mammalian cells (Igal et al., 1997) and its selective inhibition on *Cryptosporidium* both *in vitro* and *in vivo* (Guo et al., 2014). Collectively, the present study not only supports the notion that ACS enzymes can be explored as drug targets in *Giardia*, but also provides strong proof-of-concept data for the further identification of triacsin C analogs and other classes of small molecules for developing more selective inhibitors against the parasite.

## Conflict of Interest Statement

The authors declare that the research was conducted in the absence of any commercial or financial relationships that could be construed as a potential conflict of interest.

## Footnotes

1 http://www.niaid.nih.gov/topics/BiodefenseRelated/Biodefense/Pages/CatA.aspx

2 http://www.giardiadb.org

3 http://www.ncbi.nlm.nih.gov/

4 http://www.ncbi.nlm.nih.gov/cdd/

5 http://zhanglab.ccmb.med.umich.edu/TM-align/

6 http://www.idmb.tamu.edu/gtl/
