# Supplementary material for: Giardia fatty acyl-CoA synthetases as potential drug targets
Source: Front Microbiol. 2015 Jul 22;6:753. doi: 10.3389/fmicb.2015.00753 (PMC4510421; doi:10.3389/fmicb.2015.00753)
Supplement: Supplementary file 1 [file Data_Sheet_1.PDF]

Supplementary Material

Giardia fatty acyl-CoA synthetases as potential drug targets

Fengguang Guo[1], Guadalupe Ortega-Pierres[2*], Raúl Argüello-García[2], Haili Zhang[1] and Guan Zhu[1*]

[1]Department of Veterinary Pathobiology, College of Veterinary Medicine & Biomedical Sciences, Texas A&M University, College Station, Texas, USA; [2]Department of Genetics and Molecular Biology, Center for Research and Advanced Studies IPN, Mexico

***Correspondence:**

Dr. Guadalupe Ortega-Pierres, Departamento de Genética y Biología Molecular
Centro de Investigación y de Estudios Avanzados-IPN, Av. Instituto Politécnico Nacional 2508
San Pedro Zacatenco, 07360 Mexico City, Mexico
Email: gortega@cinvestav.mx

Dr. Guan Zhu, Department of Veterinary Pathobiology, College of Veterinary Medicine & Biomedical Sciences, Texas A&M University, College Station, Texas 77843-4467, USA
Email: gzhu@cvm.tamu.edu

Supplementary Figure 1. Alignment of the whole 5 *Giardia intestinalis* ACS (GiACS) protein sequences. See notes below on the annotations

Figure S1. Alignment of the 5 Giardia intestinalis ACS (GiACS) protein sequences. See notes below on the annotations

```
ACS1_GL50803_15063    1 MI-F------------PFLKPKMTPHP---TIYLNPYKSRTGLGDDPVTAPADEE---TL 41
ACS2_GL50803_9062     1 MSDF----------ICPFDAPGLDVSG---DAC-------------------------TG 22
ACS3_GL50803_21118    1 MRFQKINSAFFELKKAPFEMDRVQIADGCAFIHPSPLDTISHV---ALDEERELNPSIPV 57
ACS4_GL50803_30476    1 MT-F----------LIPFEAPEFIVDD---SIA-------------------------TI 21
ACS5_GL50803_113892   1 MSEPR--------------KPELIN--GEYHIYECPITTKSHM---PVQEDAETDTTKTI 41

ACS1_GL50803_15063   42 YAIWEKYSLLRLDELVYFHSP--LRFNAQTQT-----------IERLAYEGITYRTMRSN 88
ACS2_GL50803_9062    23 LRIMQKTTL-VTPGLRFLGTREFLRPMTAPPVLKSSKFDPPYKGSRGDFEWYSYAQVYKM 81
ACS3_GL50803_21118   58 LKLVRKAFK-RFSDKPYLGYRAGLATCV--K------------EVTDKYTYMTYGEADSY 102
ACS4_GL50803_30476   22 SRLFCQRAL-KTPRRRCLGTREFLARNDENP------------EARGDYEWYSYLDVLRM 68
ACS5_GL50803_113892  42 LQNVRATMK-RYPNEPYLGYRAGLATCV--K------------EVTDKYTYMTYGEADSY 86

ACS1_GL50803_15063   89 IERFATAFVTKFGLKPGSMVMLWSCNSPEWIITEQACNRQSMVLVPIYDSLSAHGMSYCI 148
ACS2_GL50803_9062    82 TVELALG-LQALGITKGSKVGVISTNRVEWVVLDLACIALGAVLVPIYDTQSTEEVILVA 140
ACS3_GL50803_21118  103 VTELAKV-LAKLGIGKGDRVGIFARNSPLWLLFDFACTAVGAVVVPIYDTLGAAKTSYCI 161
ACS4_GL50803_30476   69 SSELFYG-LQAIGIKQGDHAGIMSANRVEWALVDLACSALGVVTVPIYDTLSLTDTIFIA 127
ACS5_GL50803_113892  87 VTELAKV-LTKLGIGKGDRVGIFARNSPLWLLFDFACTAVGAVVVPIYDTLGAAKTSYCI 145

ACS1_GL50803_15063  149 DATRPDILVISRDKIGRLVKTLAQAVVDLSFLKAVILTTD---LEFERAIPG-------- 197
ACS2_GL50803_9062   141 NDSQISILFVAPDRLPK----WADAASRCPSVKAVIIFDD---RLDDRALVSDYYSQFAN 193
ACS3_GL50803_21118  162 NHAEVRLLVTQTELLPL----VLGIWPSCPELQRVLIIDTSISELDDQTMNNCLHKAVPS 217
ACS4_GL50803_30476  128 NDSHVTTVFLTLDTLHT----WSTAALSCPMVKTVILFDD---RYDDRWFLH---KAWAH 177
ACS5_GL50803_113892 146 NHAEVRLLVTQTELLPL----VLGIWPSCPSLQRVVLLDMTTSSLEDIKLGSQLRKAVQF 201

ACS1_GL50803_15063  198 ----------------------NQ-VEELEQLLARSGMANPRL----------------- 217
ACS2_GL50803_9062   194 SFSCPDGKTVSLAMPTHTSTVPRR-MEEFDEGDRKYGCHNPRRRTPEEQNAVEGTKSRYS 252
ACS3_GL50803_21118  218 YIEA------QQSDGASLGIDLSKLYVAGKADDSVYKFNVLSTVTSQLGALSDATDVRMA 271
ACS4_GL50803_30476  178 A-------TKIKHPLPSSVPVAYKS-FEECDADDRVDGIAFERALTPAEKAHKAGTKIRYP 230
ACS5_GL50803_113892 202 YAEV------EKLTDAELGINSNSFYKAKKTFH--FLLDSHSSTGDAASNLSDNTDLRMK 253

ACS1_GL50803_15063  218 -----------------------------------------------TDILGYVTAAVHV 230
ACS2_GL50803_9062   253 RIISTACDTASGSHGAHFYLPDKKDIPHLGHQLFTMDKGKYGQPETVQQLMKLVSGTYHQ 312
ACS3_GL50803_21118  272 NAN--IN---------------------------DSNTHIAEVINEILEK---------- 292
ACS4_GL50803_30476  231 RRL--VCNSIPSEQVDDPFTCQYTYLPS---LEVSHDLDKTGSPI---NVLHLVSYTLHG 282
ACS5_GL50803_113892 254 TAN--VS---------------------------EENTAVAAVLEKLLQCLDLQSCTDD- 283

                                                                                 v
ACS1_GL50803_15063  231 VP---------------------------------PV-----------PPTPSTISSLIY 246
ACS2_GL50803_9062   313 VTTLGRETDRYKAMFSKYAQSSATGLAGYSYLYSDDFPITSFPSLDLEVSQRDDLMTLIY 372
ACS3_GL50803_21118  293 --TQIKES-PGKISFLKNMLDEVKGLD-----YKG----------DYE-ASVDEVHTILY 333
ACS4_GL50803_30476  283 VMACGRKASAYRGVDFRKA-----------YL-SPDTLITPVYALD--LTGPDDLMTLVY 328
ACS5_GL50803_113892 284 --TLKSAT-TDKLLFLSCELSSVQGLD-----YVG----------DCP-LSMDDLHTILY 324

                        a
                        vvvvvv vv                                      c
ACS1_GL50803_15063  247 TSGTSSTPKGVIISHGALTSA-CYGYLL--TPLDFVRSGRGLHYLSYLPMAHIYERVIQA 303
ACS2_GL50803_9062   373 TSGTTGNPKGVKLSHGNVMHT-AISMQC--SRLL-PLQGQQHYTLSFLPNAHIFMRVINF 428
ACS3_GL50803_21118  334 TSGSSGNPKGVTHTHSTIQGSYTMVYGFLPY-V--SRDGGQMVTMSYLPLGHIYESAGEI 390
ACS4_GL50803_30476  329 TSGTTGNPKGVKLTQGNVLWT-SASMAE--HRVE-KRPNVQEFNLSYLPNAHIYQRVIQG 384
ACS5_GL50803_113892 325 TSGTSGNPKGVVHTQKTILGGYCMGRAFFPYRLS-HITGRITTILSYLPLGHIYEREIEH 383

                                                           cc  c          aa
ACS1_GL50803_15063  304 TGIIIGGKVYFWRGDFKSLLRDMQDVKPDILCGVPKVYTTLYQAIRSKLEAS-PLTRGWF 362
ACS2_GL50803_9062   429 VSIHSGACMGFWQGDIKMLVEDVQMLCPTFFVAVPRVLNKLFDGIMAKVSALSSFKRFVF 488
ACS3_GL50803_21118  391 LFTVRGYKIAYYSGNTKNLTTDIKLAKPTILLAVPRVLQKIYNAVMAKVEAS-LVKKAVF 449
ACS4_GL50803_30476  385 VMWYCSGATGFWCGSIKGLTNDIQALRPTIFIAVPRILNRLFEGIMASIEKLSVFRRFLF 444
ACS5_GL50803_113892 384 YCSIRGCKIAYYAGNTKNLTTDIKLAKPTILLAVPRVLQKIYNAVMVKVEAS-LVKKAVF 442

                          a
ACS1_GL50803_15063  363 HYGLK--GD---SMLQ----RFARRCLYGLVKRKIGVNFKLMISGGASLPPKVHLFVQDF 413
ACS2_GL50803_9062   489 FAAYGSRRK---ALLAGRPWPGLTNKIFSSTKALLGGRCSLCVTGSAPLSQKVGEFLRIA 545
ACS3_GL50803_21118  450 CLACKVKEYTEGSWIKTGTLPWLDNVVFKDIKAALGGHLELIVSGSSALPQEVWRFMRLC 509
ACS4_GL50803_30476  445 FLVFSTRRK---AMRRNTSWPRWTNIFFSATKALLGGRCSLCVTGSAPLSQKVGEFLHIA 501
ACS5_GL50803_113892 443 CLACKVKEYTEGSWIKTGTLPWLDNVVFKDIKAALGGHLELIVSGSSALPQEVWRFMRLC 502

                              aaaaaa
ACS1_GL50803_15063  414 FQCNLIQGYGQTETCACVTVQCESDYSVGN-CGVV-CPNMELRI----------D--P-S 458
ACS2_GL50803_9062   546 CDMEVFEGWGMSETAAHGVVQPTSTLHWGT-VGVTLDSSTKIRLTSVPDMDYLVTDKPNP 604
ACS3_GL50803_21118  510 TGARITCGYGLTETCASGLCQLPHMPYVGSPTGS-LVPFMQAKLIDKSDTCEL-SLKTHR 567
ACS4_GL50803_30476  502 CDTQVIEGWGMTETAAHGTVQSINTNHWGY-VGVTLDSSTKIRLTSVPDMDYLVTDKPNP 560
ACS5_GL50803_113892 503 TGARITCGYGLTETCLVGLRVLPHDPIEYSPAGK-LVSFMQAKLIDKSDTCEL-SLKTHR 560

                                                               a            a  a
ACS1_GL50803_15063  459 ENEVLLRGSSMFSRYYVPGETFYENIHNFPPSTEWYRSGDCGLMNE-DNKLILKDRINTN 517
ACS2_GL50803_9062   605 RGEVWIKGPSVFSGYYNDDAKTKETL----TEDGWFMTGDIGEYDRSLGELRLIDRKRGI 660
ACS3_GL50803_21118  568 VGELLLKGPGIAKEYYKMPESP------LRDADGYFHTGDLMRLND-DGSLTFVRRINMV 620
ACS4_GL50803_30476  561 RGEVWIKGPSVFSGYYNDDAKTKETL----TEDGWFMTGDIGEYDRSLGELRLIDRKGCI 616
ACS5_GL50803_113892 561 VGELLLKGPGIAKEYYKMPESP------LRDADGYFHTGDLMRLND-DGSLTFVRRINMV 613

                            ccca
ACS1_GL50803_15063  518 FKLQNGEFVCPETIELMYLQSALISAIYIPQTPNSNHLYAFVVPSESLL-AKLNVANYFS 576
ACS2_GL50803_9062   661 FKLAQGEYISATTIENAISRCKYVEQCIMHGSRFESCVLCVVVLNVDMCQKELGHAI--- 717
ACS3_GL50803_21118  621 VKSLMGEFIDICAVEDAMEKSPLFASVFVHAQSDKSFPICVAVLDKTALAHRLKC-S--- 676
ACS4_GL50803_30476  617 FKLSQGEFICTTTIENTLLQSKLIDQCFVYGDRLQSYLLAIIVPNFTELRAVLS-AT--- 672
ACS5_GL50803_113892 614 VKSLMGEFIDICAVEDAMEKSPLFASVFVHAQSDKSFPICVAVLDKTALAHRLKC-S--- 669

ACS1_GL50803_15063  577 PDQPLSLTGLHDKLVDDTATDSVHTNEQVEALGSFSVLEGKHVENHIMGLLKQKIVKDL- 635
ACS2_GL50803_9062   718 -------------------PKDVEGKLEITLQ------DPSAYP--RLKDLFMQVLQAI- 749
ACS3_GL50803_21118  677 -------------------TDDVEKAEYAAATK--------------------KILEAEA 697
ACS4_GL50803_30476  673 -------------------PGTVKDLAHLSNS---------ELV--IKEAAIITILREV- 701
ACS5_GL50803_113892 670 -------------------TDDVERPEHTDTIK--------------------SLLANEA 690

                                 c c                       a
ACS1_GL50803_15063  636 TKVHSEAGMKPFMHLKDIH-LLRREFTIENGCLTATQKLCRKKLYNQLQPEIEAMV---- 690
ACS2_GL50803_9062   750 QKTCKENSLRSFEIPKALV-VEGDPWTPENGCLTPAMKVKKINVLKRHETTLTDIYRRIN 808
ACS3_GL50803_21118  698 ASFVKRVGLKGYCVPKCFRWLFDIDWSTDQDLMTPSLKKQHRFFAKRYAAEIASMWEE-- 755
ACS4_GL50803_30476  702 DQACVARNFRPYEIPRAVI-LEHQPWTQKDESLTPALKIRRTMLTNRYKPCLSSLYHHIN 760
ACS5_GL50803_113892 691 NIFVKRAGLKGYCVPKCFRWLFDIDWSTDQELMTPSMKKQHRFFAKRYATEIASMWEE-- 748

ACS1_GL50803_15063  691 ------------------------------------------DKE 693
ACS2_GL50803_9062   809 HAGLQNVTPQGAVELYLDSLVSGPMSDTASSGFSGFSGFSRPSSK 853
ACS3_GL50803_21118  756 -----------------------------------LEGSERALAK 765
ACS4_GL50803_30476  761 KISSHKPSMQ-QIALTVIKVIDGATLSEVSTQITGASGLSEMSRQ 804
ACS5_GL50803_113892 749 -----------------------------------LQSFDRQNSK 758
```

Note: Annotation based on Conserved Protein Domain Family "LC-FACS_euk" at http://www.ncbi.nlm.nih.gov/Structure/cdd/cdd.shtml

v: acyl-activating enzyme (AAE) consensus motif (also boxed)
Comment 1: The AAE consensus motif is represented in the PROSITE PS00455 consensus sequence [LIVMFY]-X-X-[STG]-[STAG]-G-[ST]-[STEI]-[SG]-X-[PASLIVM]-[KR]
Comment 2:This motif appears to be absolutely necessary for binding of ATP and adenylate formation
Citations: PMID 12627952; PMID 8805533; PMID 12805634

c: putative CoA binding site [chemical binding site]
Comment: based on the structures of other family members with bound CoA Citation: PMID 12627952

a: putative AMP binding site [chemical binding site]
Comment: based on the structures of other family members with bound AMP Citation:PMID 12627952; PMID 9250661; PMID 16541080
